# Internal Carotid Artery Aneurysm Disguised as Pituitary Macroadenoma

**DOI:** 10.1210/jcemcr/luad076

**Published:** 2023-07-01

**Authors:** Neha Mulpuri, Hans K Ghayee, Jessica Abramowitz, Sasan Mirfakhraee

**Affiliations:** Department of Internal Medicine, UT Southwestern Medical Center, Dallas, TX 75390, USA; Malcom Randall VA Medical Center, Division of Endocrinology & Metabolism, University of Florida, Gainesville, FL 32608, USA; Division of Endocrinology & Metabolism, UT Southwestern Medical Center, Dallas, TX 75390, USA; Division of Endocrinology & Metabolism, UT Southwestern Medical Center, Dallas, TX 75390, USA

**Keywords:** pituitary adenoma, adrenal insufficiency, sellar lesion, intracranial aneurysm

## Abstract

Hypopituitarism due to an internal carotid artery (ICA) aneurysm is rare. We present a case of hypopituitarism and hyperprolactinemia due to a giant right ICA aneurysm. A 56-year-old woman with a history of primary hypothyroidism presented with fatigue, right-sided headache, and blurred vision. Magnetic resonance (MR) of the brain revealed a sellar mass measuring 3.5 × 2.2 cm involving the right cavernous sinus. Initial neurologic examination was unremarkable, and her biochemical evaluation revealed secondary adrenal insufficiency, central hypogonadism, low serum free thyroxine, and mildly elevated serum prolactin, consistent with stalk effect. Hydrocortisone therapy was started for secondary adrenal insufficiency and her levothyroxine dose was adjusted. The patient was referred to neurosurgery for surgical management of her sellar lesion. Preoperative computed tomography angiography (CTA) of the brain revealed a right ICA aneurysm that contacted the optic chiasm and displaced the pituitary gland. The aneurysm was embolized and diverting stents were placed. Repeat laboratory tests showed resolution of the patient's secondary adrenal insufficiency, normalization of serum prolactin, and an increase in serum gonadotropin concentrations to the postmenopausal range. This case highlights that not all sellar lesions are pituitary adenomas, and CTA should be performed in the evaluation of large cavernous sinus lesions to exclude ICA aneurysm.

## Introduction

We report a 56-year-old woman who presented with headache and vision changes with imaging findings initially concerning for large pituitary adenoma. However, preoperative imaging revealed a large right internal carotid artery (ICA) aneurysm as the cause of her hypopituitarism and mild hyperprolactinemia. The differential diagnoses for sellar masses include pituitary adenoma, other neoplasms, metastases, cystic lesions, vascular aneurysms/malformations, infection, and inflammatory lesions. The most common intrasellar pathology is pituitary adenoma. A retrospective review of approximately 4000 patients diagnosed with hypopituitarism at an academic center found that 7 patients (0.17%) had hypopituitarism due to an intrasellar aneurysm [1]. There are approximately 40 cases of cerebral aneurysms causing hypopituitarism described in the literature [2]. One study reported that in 5792 autopsy cases, they discovered 32 cases of coexisting pituitary adenomas and clinically silent cerebral aneurysms [3]. Preoperative diagnosis is essential for successful outcomes given that rupture of an ICA aneurysm can cause severe morbidity and/or death. Unfortunately, first-line imaging of a sellar mass—magnetic resonance (MR) brain with gadolinium contrast—does not reliably distinguish sellar aneurysm from adenoma.

## Case Presentation

A 56-year-old woman with a history of primary hypothyroidism and migraines was referred to the endocrine clinic with concern for pituitary adenoma. The patient reported a pounding, right-sided headache for 8 days with associated fatigue, nausea/vomiting, postural lightheadedness, and blurred vision (right greater than left), which prompted an urgent care visit. She denied any galactorrhea, weight loss, diplopia, or change in hat/ring size. There was no relevant family history of pituitary masses or endocrine disorders. The only home medication she was taking was levothyroxine 50 mcg daily for hypothyroidism, which she had been taking for more than a decade.

## Diagnostic Assessment

**Figure 1.**
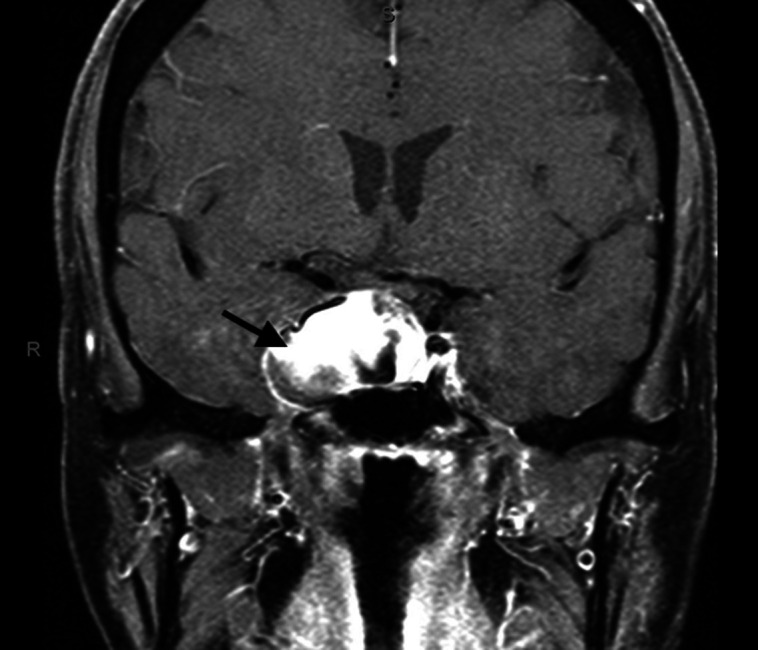
T1-weighted magnetic resonance of the brain revealed a large, heterogeneous right sellar mass (black arrow) involving the right cavernous sinus.

On physical examination, her vital signs were unremarkable and funduscopic exam revealed flat optic discs of normal color with sharp margins bilaterally. Her neurologic exam, including visual field testing, was unremarkable. Computed tomography (CT) of the head revealed a sellar mass measuring 3.5 × 2.2 cm involving the right cavernous sinus, concerning for a pituitary macroadenoma, with abutment of the right optic nerve. Subsequent initial laboratory results are listed in Table 1 and are notable for secondary adrenal insufficiency, with serum adrenocorticotropin (ACTH) 5.9 pg/mL (1.3 pmol/L) (reference range, 6-50 pg/mL; 1.3-11.0 pmol/L), cortisol less than 1 mcg/dL (<27.6 nmol/L) (reference range, 4-22 mcg/dL; 110.34-606.9 nmol/L), and dehydroepiandrosterone sulfate (DHEA-S) 5.5 mcg/dL (0.1493 μmol/L) (reference range, 16-195 mcg/dL; 0.1086-5.2923 μmol/L); central hypogonadism with inappropriately low serum luteinizing hormone (LH) 3.7 mIU/mL (reference range, 10-54.7 mIU/mL) and follicle-stimulating hormone (FSH) 9.8 mIU/mL (reference range, 23-116.3 mIU/mL); thyrotropin (TSH) 0.66 mcIU/mL (0.42-4.50 mcIU/mL) with low serum free thyroxine (T4) 0.71 ng/dL (9.139 pmol/L) (reference range, 0.8-1.8 ng/dL; 10.29-23.17 pmol/L); and elevated serum prolactin 146 ng/mL (146 μg/L) (reference range, 2-20 ng/mL; 2-20 μg/L). Serum prolactin was unchanged following serial dilutions. Dedicated magnetic resonance (MR) of the brain demonstrated a 3.5-cm heterogeneous mass that expanded the sella and extended into the right cavernous sinus suspicious for a pituitary adenoma (Fig 1).

**Table 1.** Laboratory data before and after flow diversion embolization of right internal carotid artery aneurysm

Test	Baseline	1 Mo	3 Mo	6 Mo
Serum cortisol, mcg/dL	<1 (<27.6 nmol/L)	1 (27.6 nmol/L)	7.9 (217.9 nmol/L)	12.2 (336.6 nmol/L)
ACTH, pg/mL	5.9 (1.3 pmol/L)	16 (3.5 pmol/L)	23 (5.1 pmol/L)	33 (7.3 pmol/L)
DHEA-S, mcg/dL	5.5 (0.1 µmol/L)			
LH, mIU/mL	3.7			21.3
FSH, mIU/mL	9.8			41.6
IGF-1, ng/mL	78 (10.1 nmol/L)		80 (10.5 nmol/L)	
Growth hormone, ng/mL	0.4 (1.2 mIU/L)			
Prolactin, ng/mL	146 (146 μg/L)	54 (54 μg/L)	61 (61 μg/L)	22.7 (22.7 μg/L)
TSH, mcIU/mL	0.66			
Free thyroxine, ng/mL*^a^*	0.7 (9.1 pmol/L)	1.1 (14.1 pmol/L)	1.0 (12.9 pmol/L)	1.0 (12.9 pmol/L)

Normal ranges: morning serum cortisol 5 to 23 mcg/dL (137.9-634.5 nmol/L), ACTH 6 to 50 pg/mL (1.32-11 pmol/L), DHEAS 16 to 195 mcg/dL (0.43-5.3 µmol/L), LH 10 to 54.7 mIU/mL, FSH 23 to 116.3 mIU/mL, IGF-1 40 to 217 ng/mL (5.2-28.4 nmol/L), growth hormone 0.01 to 3.61 ng/mL (0.03-10.8 mIU/L), prolactin 2 to 20 ng/mL (2-20 μg/L), TSH 0.42 to 4.5 mcIU/mL, free thyroxine 0.8 to 1.8 ng/mL (10.3-23.2 pmol/L).

Abbreviations: ACTH, adrenocorticotropin; DHEA-S, dehydroepiandrosterone sulfate; FSH, follicle-stimulating hormone; IGF-1, insulin-like growth factor 1; LH, luteinizing hormone; TSH, thyrotropin.

a Free thyroxine concentrations were measured on levothyroxine therapy.

## Treatment

**Figure 2.**
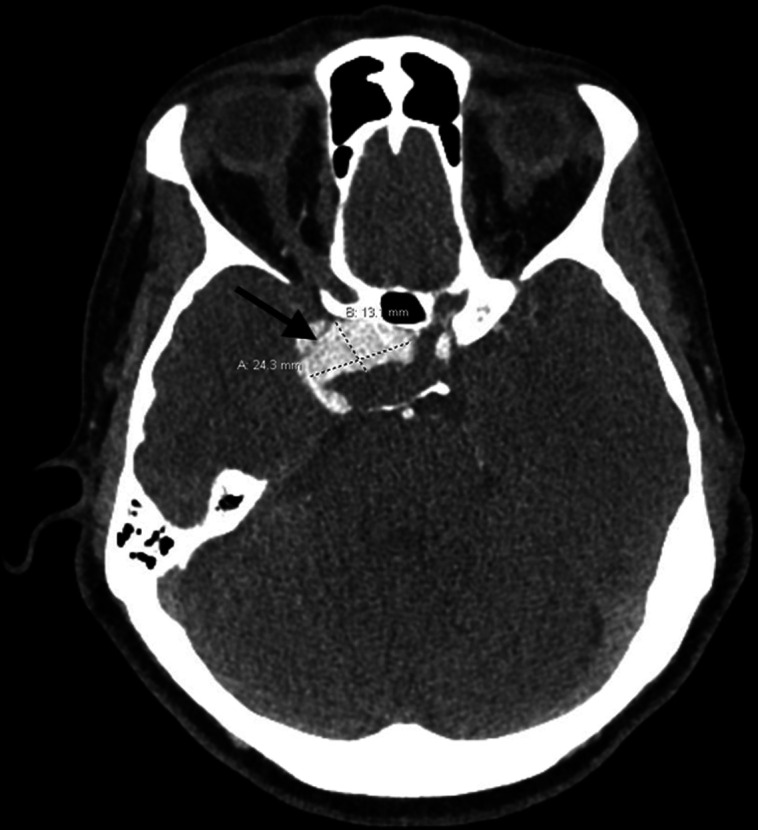
Computed tomography angiography of the brain revealed a large mass involving the pituitary fossa and right cavernous sinus concerning for a large, partially thrombosed aneurysm arising from the distal cavernous portion of right internal carotid (black arrow).

The patient was diagnosed with secondary adrenal insufficiency and started on physiologic glucocorticoid therapy with hydrocortisone 10 mg in the morning and 5 mg in the afternoon. Given her considerable fatigue, the levothyroxine dose was increased to 88 mcg daily to target free T4 in the mid-normal range. The patient was referred to the neurosurgery clinic and definitive treatment options were discussed. Transsphenoidal resection was planned, and the patient had computed tomography angiography (CTA) of the brain to better characterize the tumor and vascular anatomy. CTA brain revealed a 1.3 × 2.4 × 1.4 cm partially thrombosed right cavernous internal carotid aneurysm with mass effect on the medial aspect of the right temporal lobe, contacting the optic chiasm, and displacing the pituitary gland, without the presence of an adenoma (Fig 2). The patient was then referred to interventional radiology for endovascular embolization. She was pretreated with dual antiplatelet therapy (aspirin 325 mg daily, clopidogrel 75 mg daily). Approximately 3 months after symptoms began, the patient underwent flow diversion embolization of right ICA aneurysm with 3 telescoped stents deployed in a retrograde fashion with no complications.

## Outcome and Follow-up

**Figure 3.**
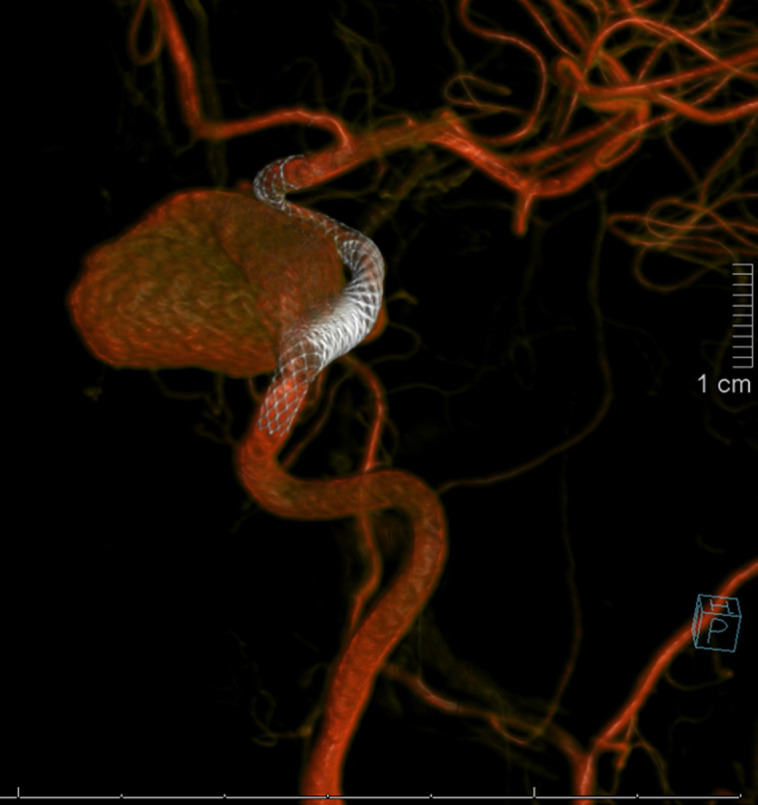
Magnetic resonance angiography of the brain demonstrating pipeline stent embolization of giant right cavernous internal carotid artery aneurysm with patent flow proximal and distal to stent.

The patient noted improved headaches after the procedure. Postprocedure MR angiography of the brain revealed no clear flow within the aneurysm with patent flow proximal and distal to stent placement (Fig 3). Three months after flow diversion embolization, biochemical evaluation revealed improved secondary adrenal insufficiency with serum ACTH 23 pg/mL (5.065 pmol/L) (reference range, 6-50 pg/mL; 1.32-11.01 pmol/L) and cortisol 7.9 mcg/dL (217.93 nmol/L) (reference range, 4-22 mcg/dL; 110.345-606.90 nmol/L) and reduced serum prolactin 61.3 ng/mL (61.3 μg/L) (reference range, 2-20 ng/mL; 2-20 μg/L). Consideration was made for ACTH stimulation testing to assess for hypothalamic-pituitary-adrenal axis recovery; however, given the patient's distance from clinic, it was elected to continue her on physiologic glucocorticoid therapy. Three months later, the patient's basal serum cortisol concentration improved to 12.2 mcg/dL (336.55 nmol/L) (reference range, 4-22 mcg/dL; 110.345-606.90 nmol/L) with ACTH 33 pg/mL (7.27 pmol/L) (reference range, 6-50 pg/mL; 1.32-11.01 pmol/L), and hydrocortisone was discontinued. At this time, the patient's serum prolactin normalized at 22.7 ng/mL (22.7 μg/L) (reference range, 2-20 ng/mL; 2-20 μg/L) and serum gonadotropins increased to postmenopausal concentrations: LH 21.3 mIU/mL (reference range, 10-54.7 mIU/mL) and FSH 41.6 mIU/mL (reference range, 23-116.3 mIU/mL).

## Discussion

Intracranial aneurysms have a prevalence of 3% to 5%, though only 1% to 2% of those are found near the pituitary gland [2]. Cerebral aneurysms that lead to hypopituitarism are even rarer, accounting for less than 0.2% of cases, and there are approximately 40 reported cases in the literature [2]. Given their rarity, ICA aneurysms are rarely considered in the differential of cavernous sinus lesions. A systematic review of 40 cases of intrasellar aneurysms found that the most common symptoms included headache (61%), visual field cuts/decreased visual acuity (61%), endocrinopathies (57%), symptomatic hyponatremia (21%), and cranial nerve paresis (other than optic nerve) (18%). The most common endocrine abnormalities associated with sellar aneurysms included hyperprolactinemia (90%), gonadotropin deficiency (82%), ACTH deficiency (70%), and TSH deficiency (60%) [2]. Intrasellar aneurysms may result in comparable endocrine dysfunction as pituitary macroadenomas [2].

The patient described here presented with headache and blurred vision, and biochemical evaluation revealed secondary adrenal insufficiency, central hypogonadism, low serum free T4, and mildly elevated serum prolactin. The exact mechanism by which sellar aneurysms cause hypopituitarism is not completely clear. Two possibilities include aneurysm compression of the hypothalamus, disrupting pituitary activating/inhibiting factors, and/or a destructive effect of the enlarging aneurysm on the pituitary gland itself [4]. Elevated serum prolactin can occur due to stalk effect causing a reduction of dopaminergic inhibition. This typically presents with mild to moderate prolactin elevation (generally 30-200 ng/mL) (30-200 μg/L) (reference range, 2-20 ng/mL; 2-20 μg/L) though very high prolactin concentrations from stalk effect have been noted previously [5]. In our case, the patient's serum prolactin concentration normalized 6 months after endovascular intervention of her right ICA aneurysm.

As this case demonstrates, it can be difficult to distinguish between a pituitary adenoma and sellar aneurysm based on biochemical and/or imaging findings. MR of the brain with gadolinium contrast is the first-line imaging for a sellar mass. Unfortunately, the degree of gadolinium enhancement does not distinguish one type of sellar mass from another. However, differentiating these diagnoses is vital given the differing management strategies. MR of the brain can demonstrate a “halo appearance” produced by the 2 dural layers encasing the cavernous portion of carotid artery aneurysms, which can help distinguish an aneurysm from an adenoma. However, in this case, there were no specific findings on routine MR of the brain that helped with such a distinction. There have been several reported cases of transsphenoidal surgery of pituitary adenomas leading to rupture of internal carotid aneurysms and subarachnoid hemorrhage or carotid-cavernous fistulae, though the incidence is rare [6, 7]. Given this risk, we recommend preoperative studies of intracranial vasculature, such as CTA, to characterize large cavernous sinus lesions prior to transsphenoidal surgery to exclude ICA aneurysm. Intracavernous aneurysms generally have a benign course, though serious complications such as meningeal hemorrhage (1.7%) or carotid-cavernous fistulae (8%) can occur [8]. Endovascular therapeutic options are the standard of care. Recovery of pituitary function after endoscopic management of an ICA aneurysm is rare and correlates with residual pituitary function before treatment. In one case, a patient with hypopituitarism in the context of a large ICA aneurysm recovered pituitary function 10 months after the procedure [9]. Our patient ultimately recovered hypothalamic-pituitary function 6 months after endovascular intervention.

## Learning Points

- Not all sellar lesions are pituitary adenomas. ICA aneurysms are rare but should be included in the differential of sellar lesions, particularly those involving the cavernous sinus
- Preoperative studies of intracranial vasculature are recommended in patients with large cavernous sinus lesions prior to transsphenoidal surgery
- Reassessment of the hypothalamic-pituitary axes should be performed after endoscopic management of ICA aneurysms

## Contributors

All authors made individual contributions to authorship. N.M., H.G., J.A., and S.M. reviewed and approved the final draft. S.M. was involved in the diagnosis and management of this patient. N.M., H.G., J.A., and S.M. were involved in manuscript writing. N.M. and S.M. were involved in manuscript submission.

## Data Availability

Data sharing is not applicable to this article as no data sets were generated or analyzed in the current study.

## Abbreviations

ACTH: adrenocorticotropin
CTA: computed tomography angiography
DHEAS: dehydroepiandrosterone sulfate
FSH: follicle-stimulating hormone
ICA: internal carotid artery
IGF-1: insulin-like growth factor 1
LH: luteinizing hormone
MR: magnetic resonance
T4: thyroxine
TSH: thyrotropin
